# O11.2. CHANGES IN PSYCHOPATHOLOGY PREDICT CHANGES IN WORKING ALLIANCE IN FIRST EPISODE PSYCHOSIS

**DOI:** 10.1093/schbul/sby015.262

**Published:** 2018-04-01

**Authors:** Marianne Melau, Nikolai Albert, Merete Nordentoft

**Affiliations:** 1 Mental Health Centre Copenhagen, Copenhagen University Hospital; 2 Mental Health Centre Copenhagen

## Abstract

**Background:**

The cooperative and dynamic relationship between patients and therapist known as Working Alliance, has in two meta-analysis shown to be an important factor for positive outcome in psychotherapy regardless the modality of therapy. Studies investigating the association between working alliance and outcome conducted in cohorts of patients with mental illness treated in a case manager setting has reported an association between a strong working alliance and reduced symptom severity, better social function, adherence to psycho-social treatment.

For this study, we used data from a trial testing the effect of five years of specialized early intervention (SEI) compared to two years of SEI for patients diagnosed with first episode of schizophrenia spectrum disorder. We aimed to study the effect of the intervention on the working alliance and the change in working alliance as a dynamic factor in the two treatment conditions from baseline to follow-up.

When extending specialized early intervention from two to five years’ vs transferring to treatment as usual, we hypothesized a change in working alliance and psychopathology favoring the patient in the extended SEI group.

**Methods:**

Participants were recruited from SEI teams (OPUS) in Denmark. All newly diagnosed within the schizophrenia spectrum (ICD-10, F2), age between 18 and 35. Participants were included 1 ½ year after initiation of SEI treatment (baseline) and followed up 5 years after initiation of treatment. At both assessments participants were examined with a comprehensive assessment battery including working alliance, psychopathology, social function, cognitive function, adherence to medication and client satisfaction. Assessors were blind to treatment allocation. The primary outcome, working alliance inventory (WAI), was assessed by self-assessment.

A change score was calculated by subtracting the baseline score from the follow-up score. Multivariable linear regression analyses were conducted, corrected for the baseline value of the independent and dependent variable.

**Results:**

Of the 289 participants who attended the follow-up interview 258 (89%) had completed the WAI at baseline and follow-up. Participants who were randomized to prolonged SEI had a stable WA from baseline to follow-up, while participants who were randomized to TAU had a mean drop in WA over the same period.

Change in WA was associated with change in negative-, psychotic-, and disorganized symptoms dimension, and social function in the extended OPUS group. In the TAU group, we found that change in WA were negatively associated with change in cognitive function measured with BACS. In both groups, there were an association between the change in WA and change in client satisfaction.

**Discussion:**

This indicates that those participants’ who continued the extended SEI treatment maintained their experiences of a strong WA with their case manager, while those participants who were transferred to TAU experiences a lower degree of WA with their case manager compared to their time in SEI treatment. Furthermore, the participants who increased on their cognitive functioning were less likely to assess WA positively if they were transferred to TAU.

